# Mr. Asquith and Nurse Registration

**Published:** 1913-05-03

**Authors:** 


### MR. ASQUITH AND NURSE REGISTRATION.

To those who know most about the registration of nurses' controversy, its history and the proceedings in the past, the deputation to the Prime Minister gave cause for some amusement. Why, it is wondered, were most of the former orators who have made themselves so prominent, dumb in the presence of Mr. Asquith? Why, too, was not a single member of Parliament backing the Bill present among the speakers? and why was Dr. W. A. Chapple put up to introduce the deputation? If we may judge by the statement he made about the London Hospital, it would appear as if someone who knew better had presumed upon his being new to the subject and had fed him with stale bread. A story some twenty-eight years old in support of a Bill now before the House of Commons is scarcely suitable for presentation by a deputation to the Prime Minister of England.

On the question of nurse registration our position is one of entire independence. We have for many years resisted the attempt made by certain interested parties to represent themselves as the nursing profession in these islands and to force their will upon every British nurse. The story of these attempts is still fresh, and those who are interested will find it accurately set forth in the pamphlet entitled "Make-Believe in British Nursing," published by The Scientific Press, Southampton Street, Strand. We have held, and still hold, that nurse registration can never pass unless it gives adequate representation and recognition to those who are responsible for the training and education of nurses in these islands.

The present Bill contains no recognition of the great nurse-training schools and other institutions where some 9,000 probationers are being trained which certify some 3,000 nurses every year. Yet these institutions have spent hundreds of thousands of pounds in the education, training, and building up of our present system of nurses for the sick, and they alone command the knowledge, experience, and means whereby an adequate standard of knowledge for professional nurses can be maintained and enforced. Who else but the authorities of these great schools of nursing would be consulted first by a statesman who contemplated legislation to set up a standard of training and to provide for the adequate examination and registration of trained nurses? No central body appointed by the State which ignores the whole of these nurse-training schools would ever command the support or secure the confidence either of the public or of the most capable, highly-trained, and best British nurses. That is why Mr. Munro Ferguson's Bill, as it stands, must fail to pass and deserves to fail. It altogether ignores the nurse-training schools and those actively engaged in the training and education of nurses in this country, and places the control of the proposed system of registration at the outset largely in the hands of past matrons and representatives of self-elected societies which have no part whatever in the education and training of nurses.

Some of the speakers at the deputation appear to regard registration as a panacea for existing evils. Sir Victor Horsley declared, for instance, that "the medical profession knew very well the advantages they had gained by registration, and they wanted to see the same advantages in the nursing profession." In the same daily papers which recorded his speech, Sir Victor had evidence that under the National Insurance Act the medical profession may, through the Insurance Committees, be placed in competition with herbalists, Christian Scientists, and other unqualified persons. Registration evidently does not give protection to the medical profession from unqualified persons, nor would it protect nurses from these evils, though its advocates maintain to the contrary. The truth is, and it should be widely recognised, that any Bill for the protection of nurses to succeed must cover a much wider field than the present one. We must have united action by taking into counsel those who are and have been responsible for the education and training of nurses from the commencement. Mr. Asquith, as a statesman, recognised this fact, and rightly insisted that in the present state of public opinion on the subject it was impossible for the Government to give facilities to the present Bill.

The only possible Bill must be a knowledgeable, statesmanlike, and impartial measure, wide enough to embrace every side of the question and to protect and uphold every interest involved.

